# Stem Cell-Derived Beta Cells for Treatment of Type 1 Diabetes?

**DOI:** 10.1016/j.ebiom.2014.10.018

**Published:** 2014-10-29

**Authors:** Robin Goland, Dieter Egli

**Affiliations:** a Naomi Berrie Diabetes Center, College of Physicians and Surgeons, Columbia University, New York, NY 10032, USA; b Department of Medicine, College of Physicians and Surgeons, Columbia University, New York, NY 10032, USA; c Department of Pediatrics, College of Physicians and Surgeons, Columbia University, New York, NY 10032, USA; d The New York Stem Cell Foundation Research Institute (NYSCF), New York, NY 10032, USA

The discovery of insulin more than 90 years ago transformed T1D from a fatal disease to a manageable condition. Improvements in insulin formulation and development of devices for patients to deliver insulin and measure blood sugar have combined to produce one of the most remarkable success stories in drug and device development in the 20th century. Life expectancy in T1D has increased dramatically and many patients, particularly those who are able to sustain the demanding and costly self-care regimen that good glucose control requires, live long and healthy lives. But T1D remains a very serious chronic illness – commonly beginning in childhood – a disease that imposes a huge burden on the patients themselves, as well as their families and on society as a whole. Furthermore, despite advances in insulin delivery and glucose monitoring, it is extremely challenging to achieve long-term glucose control, resulting in significant risks for acute and chronic complications. In addition, the incidence of T1D continues to increase, especially in the very young. In order to reduce the human suffering related to T1D as well as the large financial costs of T1D and its complications, efforts at curing T1D in those who have already been diagnosed, and preventing it for the future, are of paramount importance.

A cure for T1D will likely involve the use of replacement beta cells, the insulin-producing cells of the pancreas that are lost in the autoimmune assault that is a hallmark of the disease. Beta cells both produce insulin and sense glucose and calibrate the delivery of appropriate amounts of insulin without causing hypoglycemia or permitting hyperglycemia, a remarkable process occurring reliably – and automatically – throughout a lifetime in a person without diabetes.

Cadaveric beta cells are already in clinical use. Islet cell transplantation or pancreatic transplantation can, in principle, cure T1D — although long-term success rates with current procedures are not optimal. More than 1000 pancreas transplants are performed each year in the USA, most for T1D (Gruessner, 2011). Worldwide, fewer than 100 islet transplants are performed yearly (Barton et al., 2012). Life-long immunosuppressive therapy is required to prevent the recurrence of T1D from autoimmunity and to prevent allo-rejection. The need for immunosuppression, along with the scarcity of donor islets, restricts current use of cadaveric transplantation to only a small subset of T1D patients, such as those who are receiving immunosuppression for another indication, such as a kidney transplant.

Another potential source of beta cells that could be used in diabetes treatment are embryonic stem cells. Baetge and colleagues reported preliminary success at beta cell differentiation — they showed that exposing embryonic stem cells to a series of molecules participating in pancreatic development gave rise to insulin-producing cells in vitro (D'Amour et al., 2006). These cells were not functionally mature: they co-expressed glucagon in addition to insulin, and failed to increase insulin secretion in response to high glucose ambient levels. But remarkably, when these immature cells were transplanted into mice, mature beta cells formed — cells that could maintain normal glucose levels after ablation of the mouse's intrinsic pancreatic beta cells (Kroon et al., 2008). These pioneering studies are now the basis for an ongoing clinical trial that will determine whether immature stem cell-derived pancreatic cells will yield insulin-secreting beta cells when transplanted into human subjects with T1D.

The recent finding by Douglas Melton and colleagues, as well as Rezania and colleagues constitutes further progress and suggests that in vitro generation of more mature beta cells from stem cells is possible in vitro before transplantation. Using a complex, multi-stage protocol, beta cells that are very similar to the cells in the human pancreas have now been generated (Pagliuca et al., 2014, Rezania et al., 2014). Unlike earlier versions, these beta cells express insulin, but not other pancreatic hormones, respond to repeated glucose stimulation with increased insulin secretion, and contain mature insulin granules by electron microscopy. And diabetic mice transplanted with these cells show normal blood glucose levels within several weeks, instead of the months required for functional maturation of beta cells in the earlier experiments. Differentiation from pluripotent stem cells can be performed in large volumes of suspended cells, allowing the production of amounts of cells (about half to a billion cells per transplant) that would be required for the treatment of human subjects. This work helps address one critical obstacle in the use of beta cells for cell replacement therapy for diabetes in the future, the availability of sufficient numbers of mature beta cells for transplantation. This report was met by understandable excitement by many in the diabetes field, including T1D patients and their families living every day with this challenging condition.

The most immediate use of stem cell-derived beta cells is in research. These cells are useful for investigation into the mechanisms of beta cell failure, as demonstrated in studies of monogenic diabetes (Shang et al., 2013). These proof-of-principle studies showed that these stem cell-derived beta cells show molecular phenotypes that recapitulate those seen in the patient. Stem cell-derived beta cells may also be applied to studies of T1D in humanized mouse models of autoimmunity. Such mice could prove critical for the testing of therapies to prevent beta cell destruction.

Use of cell based therapies for T1D will still need to overcome or bypass autoimmunity. Though we can likely prevent allo-rejection by utilizing patient-specific stem cell-derived beta cells that are generated from the T1D patient receiving the transplant (Maehr et al., 2009, Yamada et al., 2014), auto-immunity and T1D would probably recur by virtue of ongoing immune assault on molecular components of the beta cell (Tyden et al., 1996). The need for immunosuppressive drugs would again limit beta cell transplantation, even with stem cell-derived beta cells, to a small subset of patients, until the autoimmunity can be addressed. The use of encapsulation – an immune-protective membrane encapsulating the transplanted beta cells – is one possible approach under study. The durability of such transplants is unknown.

The development of personalized cell replacement therapy for diabetes is a formidable challenge. The complexity is daunting and success will require effective collaboration between experts in fields as diverse as tissue engineering, immunology, stem cell research and clinical care. Experimental treatments will need to be continuously evaluated against the efficacy and safety of current clinical care, a high standard to match. As unsolved questions regarding safety and efficacy of cell-based treatments for T1D are related to the immune system, beta cell replacement for patients with non-immune mediated diabetes, such as those with maturity onset diabetes of the young (MODY) or cystic fibrosis, may provide clinical settings in which experience with these strategies can be gained. Though success is uncertain, it is a goal worth pursuing, with potentially transformative effects on medicine, replacing life-long diabetes disease management, with a cure using the patient's own cells.
